# Extracellular vesicles as viral countermeasures: dampening of oscillations and reduction of extinction risk

**DOI:** 10.1093/femsec/fiaf030

**Published:** 2025-04-12

**Authors:** Ferdi L Hellweger

**Affiliations:** Water Quality Engineering, Technical University of Berlin, 10623 Berlin, Germany

**Keywords:** countermeasures, extracellular vesicles, model, virus

## Abstract

Microbes produce extracellular vesicles (EVs, tiny membrane enclosures) that can transport some “cargo” (signaling molecules, proteins/enzymes, toxins, and nucleic acids) away from themselves or to other cells. EVs have also been shown to adsorb virus (phage) particles and inhibit infection, so another potential function is to serve as decoys for virus infection. However, the fitness benefit has not been explored quantitatively. Here, three existing mathematical models are extended to include EVs and parameterized based on literature. Simulations include a number of environments (lab culture and ambient), conditions (equilibrium and oscillating, i.e. predator–prey cycles), and bacteria (including enteric *Escherichia coli* and marine *Prochlorococcus*). Hosts invest, on average, ∼10% of resources into EV production. The models predict that producing EVs typically results in relatively minor increases in average host concentration (average ∼4.3% of log concentration). However, under oscillating conditions, EVs can substantially dampen and, in most cases, completely eliminate fluctuations, thereby increasing the minimum concentration and reducing extinction risk. These results provide insights into the fitness benefit of EVs as viral countermeasures, and they constitute a starting point for including EVs in ecosystem models.

## Teaser

Several existing mathematical microbe-virus models were extended to include extracellular vesicles (EVs), parameterized based on the literature, and used to explore the ecological role of EV production.

## Introduction

Microbes are important players in all ecosystems, from the human gut to the global ocean, and understanding their ecology is an important scientific and societal endeavor. How do interactions with nutrients, parasites, predators, and viruses affect the fitness of a microbe? How much carbon flows along the links of these complex food webs? Models are important tools for research and management in this arena. They can be used in a scientific context to interpret field observations and test hypotheses, or for management to answer “what if” questions, e.g. what would happen in a warmer climate. However, existing microbial ecosystem models are missing a potentially important component: vesicles.

Gram-negative bacteria produce outer-membrane vesicles [OMVs, here referred to as extracellular vesicles (EVs)]. They are small (20–250 nm, versus *Escherichia coli* cell ∼1.5 μm) spherical buds of the outer membrane that are released into the extracellular environment. The EV production rate can change with various factors such as temperature, stress, etc. They can be filled with various compounds (cargo), such as signaling molecules, proteins, DNA, etc., and serve numerous functions, including delivering virulence factors to target cells, helping with nutrient acquisition, cell-to-cell communication, and others (Schwechheimer and Kuehn 2015).

Since the surface of EVs is derived from that of the cell, it is intuitively expected that viruses also will attach there and get neutralized, and many experiments have shown that (Manning and Kuehn 2011, Biller et al. 2014, Kharina et al. 2015, Reyes-Robles et al. 2018, Stephan et al. 2020, Augustyniak et al. 2022, Silva et al. 2022, Li et al. 2024). For example, Manning and Kuehn (2011) found that *E. coli* EVs irreversibly bind T4 viruses and reduce infection efficiency. Similarly, Reyes-Robles et al. (2018) showed that *Vibrio cholerae* EVs adsorb viruses, inhibit infection, and that it was receptor-specific (i.e. specific membrane proteins need to be on the EVs). Biller et al. (2014) found that EVs from the open ocean cyanobacteria *Prochlorococcus* adsorb virus PHM-2. Further, EV production by *E. coli* has been shown to be induced by viruses (Loeb 1974, Loeb and Kilner 1978, Schatz et al. 2017, Mandal et al. 2021, Silva et al. 2022). These observations suggest that EVs can function as “decoys”, akin to infrared flares used by aircraft as countermeasures against heat-seeking missiles. That is, cells produce EVs, viruses adsorb to the EVs, and those are then no longer able to infect the cells. However, there are also observations where EVs aid the virus by signaling cells to increase their infectivity or transferring receptors—and thus virus susceptibility—from one cell to another (Schatz et al. 2017, Tzipilevich et al. 2017).

If EVs are effective viral countermeasures, then they are part of the extensive battery of mechanisms used by bacteria to defend against viruses, including preventing attachment by altering or hiding receptors, blocking DNA entry, destroying virus DNA, and interrupting the infection cycle at some other downstream point (Labrie et al. 2010, Bernheim and Sorek 2020). Conceptually, EVs are inherently effective because they do not interfere with other cellular mechanisms, as, e.g. modifying receptors does, and always reflect the infectivity of the host, which may change due to co-evolution/arms race with the virus.

Are EVs effective viral countermeasures under ambient conditions? If EVs would serve no other function, then the answer would be trivial. Why would cells produce them if they were not beneficial? However, since EVs serve many other functions (e.g. delivery of some cargo, see above), their ability to adsorb viruses may be an accidental and unimportant side effect? Another question is: Do EVs significantly change the carbon flux in ecosystems? Answering these questions requires quantitative analyses, i.e. modeling using realistic conditions and parameters. To my knowledge, this has not been explored, and there is no ecological model incorporating EVs.

Here, the fitness benefit of EVs as viral countermeasures is quantitatively explored using three existing mechanistic mathematical models. Minimal modifications to the models are performed to include the production of EVs and viral adsorption. Parameterization of the original model is generally retained, and new parameters for EVs are based on literature and theory. To explore the effect of EVs on the host fitness and carbon flux, simulations with and without EVs are performed, and the results are compared. Constant boundary conditions (e.g. dilution rate, inflow substrate concentration) are used, where different values may result in equilibrium or oscillating conditions (i.e. predator–prey cycles). The models only consider the viral inactivation function of EVs. Other uses, e.g. delivery of some cargo to other cells, which may serve an additional biological role and benefit, are ignored. This way the analysis does not quantify the entire fitness benefit of EVs, but it allows for the isolation of the effect of the decoy mechanism. Further, the analysis is restricted to suspended/planktonic populations, and attached/biofilm populations are not considered.

## Methods

### Models used

The model of Levin et al. (1977) (L77) includes state variables for resource, host, infected host, and virus. A distinguishing feature of the model is that it explicitly accounts for the latent period (the time from infection to burst). The model was parameterized for *E. coli* and virus T2, and compared to laboratory data.

Miki et al. (2008) (M08) presents several model variants, and here the most complex and realistic scenario is used. This includes two groups with organic carbon, uninfected bacteria, bacteria infected by lytic virus, bacteria infected by lysogenic virus, resistant bacteria, and virus. The groups are independent, meaning the virus from one group does not infect bacteria from the other group. Finally, one protozoan state variable grazes on the bacteria from both groups. The model includes infected cells, but the latent period is simulated in a simplified manner by specifying a first-order lysis rate.

The model of Weitz et al. (2015) (W15) includes state variables for heterotrophs, cyanobacteria, and eukaryotic autotrophs, each with corresponding viruses, and zooplankton, organic, and inorganic N concentration. Unlike the other models, this model does not consider the latent period, meaning viral infection is simply a second-order reaction between cells and viruses, resulting in the loss of cells and gain of viruses. The model is parameterized for the ocean euphotic zone.

### Code modifications

The models were modified by introducing EVs, including their production and adsorption of viruses. The details of the modifications vary among the models (see supplementary data) but generally follow these simplified/reduced mass balance equations for cell/host (*H*, no./L), virus/phage (*P*, no./L), and EV (*E*, no./L):


(1) \begin{eqnarray*}
\frac{dH}{dt} = \frac{\mu }{\color{red}{1 + {f_R}\,\, {n_{EV}}}}\mathrm{\,\,}H{\mathrm{\,\,}} - \frac{Q}{V}{\mathrm{\,\,}}H - {k_I}{\mathrm{\,\,}}H{\mathrm{\,\,}}P
\end{eqnarray*}

(2) \begin{eqnarray*}
\frac{{dP}}{{dt}} = \left( {b - 1} \right){\mathrm{\,\,}}{k_I}{\mathrm{\,\,}}H{\mathrm{\,\,}}P - \frac{Q}{V}{\mathrm{\,\,}}P\ {\color{red}{- {f_A}{\mathrm{\,\,}}{k_I}{\mathrm{\,\,}}E{\mathrm{\,\,}}P}}
\end{eqnarray*}

(3) \begin{eqnarray*}
\color{red}{{\frac{{dE}}{{dt}} = {n_{EV}}\frac{\mu }{{1 + {f_R}{\mathrm{\,\,}}{n_{EV}}}}{\mathrm{\,\,}}H - \frac{Q}{V}{\mathrm{\,\,}}E - {f_A}{\mathrm{\,\,}}{k_I}{\mathrm{\,\,}}E{\mathrm{\,\,}}P}}
\end{eqnarray*}

The modifications are shown in red text and are described subsequently. The host concentration increases with first-order growth (growth rate, *μ*, 1/day) and decreases with outflow (*Q*/*V*, 1/day; flow rate, *Q*, L/d; reactor volume, *V*, L) and viral lysis (*k_I_ P*, 1/day; infection rate, *k_I_*, L/no./day). The latent period is not considered here. The virus increases with first-order viral lysis (*b k_I_ H*, 1/day; burst rate, *b*, no.) and decreases with outflow.

The host growth rate decreases based on the number of EVs produced (*n*_EV_, EVs/cell/generation) and the resource fraction required to produce one EV (*f_R_*). The virus has an additional loss due to EV adsorption (*f_A_ k_I_ E*, 1/day), where the adsorption rate for EVs differs from that of hosts by a factor (*f_A_*). The EV concentration increases by production and decreases due to outflow and viral adsorption. The last term is akin to super-infection exclusion, which does not really apply to nonliving EVs. However, considering the small size of EVs, it is reasonable to assume only one virus can adsorb to it, which is what this term in the model represents. Regulation of EV production is included in a simplified manner by making the number of EVs produced a function of the virus infection rate (ratio of infection to growth rates, details in supplementary data).

### Parameterization

Existing parameters were generally adopted from the original publications without changes. However, an additional parameterization for the L77 model for *Prochlorococcus* (*Pro*) was developed. Also, in some cases, parameters were adjusted slightly to obtain oscillating conditions. For example, for M08, the growth rate of uninfected bacteria was increased by a factor of two (details in supplementary data).

For EVs, the revised model only requires three fundamental parameters that are estimated directly from the literature, i.e. without calibration. This includes the EV production rate (*n*_EV_), resource fraction (*f_R_*) and adsorption rate fraction (*f_A_*). *f_R_* and *f_A_* depend on the size of cells, EVs, and viruses, which are estimated from the literature. For example, for *E. coli*, 1.5 μm, 75 nm, and 49 nm are used (see supplementary data; Loferer-Krößbacher et al. 1998, Milo and Phillips 2015). For some models there are additional parameters. For example, for the M08 model, the EV decay rate had to be specified, which was taken to be the same as for the virus.

The EV production rate (*n*_EV_) can be estimated from laboratory experiments. For example, Bonnington and Kuehn (2014) summarized literature for *E. coli* and *Pseudomonas aeruginosa* and concluded that 1% of membrane material is packed into EVs. Assuming spherical shapes, *d*_cell_ = 1.5 μm and *d*_EV_ = 75 nm (see supplementary data), this corresponds to a net production rate = 4.0 (= 0.01 × (*A*_cell_/*A*_EV_) = 0.01 × (*d*_cell_/*d*_EV_)^2^). For *Prochlorococcus* (*Pro*), net production rates of 3.5 (2–5) were observed in batch culture (Biller et al. 2014). These observations are for uninfected cells, and viruses have been found to increase EV production. For *E. coli*, Loeb (1974) and Loeb and Kilner (1978) observed a release rate by infected cultures ∼36 and 9 (average 23) times higher, respectively. Silva et al. (2022) found an upregulation by a factor of 2.9 for freshwater bacteria. Here, the upregulated release rate is taken as 13 (average of 2.9 and 23) times higher. For example, for *Pro*, the EV production rate ranges from a minimum of 3.5 to an upregulated maximum of 44. No observations for *Pro* EV production in the presence of viruses are available, but the production rate has been observed to change as a function of light and temperature with similar magnitudes (Biller et al. 2023).

The resource fraction (*f_R_*) accounts for the material and energy the cell spends to make one EV, relative to that for one cell. This depends on the relative size and composition of EVs, where the latter is likely to differ due to a higher surface-to-volume ratio and cargo. Also, there is energy associated with the formation of EVs, like membrane bending and other factors, but the net effect, costs versus drivers, may be positive or negative (Stachowiak et al. 2013). Here, only the effect of size is considered. Specifically, the fraction of resources required for membrane and other parts (Ortega-Arzola et al. 2024) is scaled by the surface area and volume fractions. For example, for *Pro*, the resource fraction (*f_R_*) is 5.4e−3 (details in supplementary data). For an uninduced production rate of 3.5 EV/cell/generation, that means 1.9% of resources are spent on EVs.

The overall adsorption rate of viruses to cells or EVs depends on (i) encounter and (ii) attachment. For diffusive encounters, which dominate the sub-micrometer scale, the encounter rate can be estimated from the diffusion coefficients and particle radii (Słomka et al. 2023), where the former also depend on the radii. The diffusion rate is inversely proportional to size, whereas the encounter rate is proportional to size. For equal-sized particles (e.g. cell–cell versus virus–virus contact), these factors cancel out, but not for unequal-sized particles, and in this case, the virus-EV encounter rate is smaller than the virus-cell encounter rate. This calculation does not consider motility, which would further increase the encounter rate for the motile host compared to the immotile EV. The overall adsorption rate is lower, i.e. not all encounters result in attachment, due to near-field interactions, e.g. surface charge, presence of matching receptors (Hicks et al. 2023), and the orientation of the phage relative to the cell or EV. The effect of electrostatic repulsion and Van der Waals attraction forces can be estimated from DLVO theory, which predicts the corresponding forces are proportional to particle size and the resulting “stability ratio” is proportional to the square of particle size (Elimelech et al. 1995, Stumm and Morgan 1996). That means smaller particles (here virus and EV) have a lower stability ratio and higher attachment probabilities than larger particles (here virus and cell). This effect counteracts that of the lower encounter rate. Other near-field factors, e.g. receptor lock-key interaction, may be different if the composition of cells and EVs differs, but here they are assumed to be the same. In summary, the difference in overall adsorption rate is solely due to differences in encounter rates and attachment probability, which are derived from size, and this is quantified by the adsorption rate fraction (*f_A_*). This is convenient as the infection rate for the hosts (*k_I_*), which is available from the existing models, can simply be scaled by this factor for EVs. For example, for *Pro*, the encounter rate and attachment probability factors for virus-EV are 0.28 (lower) and 2.4 (higher), respectively, compared to virus-cell. The overall adsorption fraction (*f_A_*) is 0.66.

## Results and discussion

### EVs dampen and eliminate oscillations in L77 model

**Figure 1.**
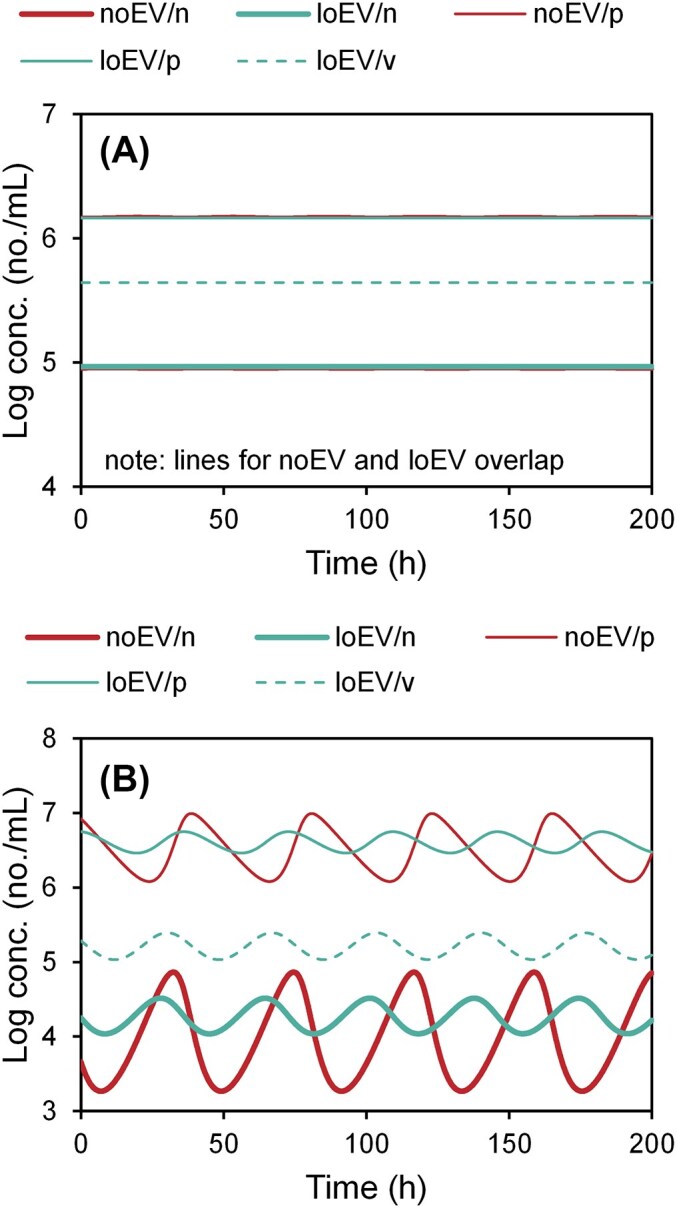
Example results from L77 *E. coli* model. Time series of host (*n*), virus (*p*), and EV (*v*) for models without (noEV) and with constant low (loEV) EV production. (A) Equilibrium. *ρ* = 0.44 1/h, *C* = 12 μg/ml. (B) Oscillating. *ρ* = 0.11 1/h, *C* = 5.0 μg/ml. Results for other model variants: L77/ *Pro* = Supplementary Fig. S3, M08 = Supplementary Fig. S5, and W15 = Supplementary Fig. S6.

The L77 *E. coli* model can predict oscillation to extinction, stable oscillations, stable equilibrium, and no equilibrium, depending on the environmental conditions (inflow resource concentration, dilution rate). In all cases, temporally constant boundary conditions are used, and oscillations are due to Lotka–Volterra-type predator–prey cycles. Stable equilibrium and stable oscillating conditions correspond to host survival, and two examples are presented (Fig. 1).

For equilibrium conditions (Fig. 1A), the host concentration increases slightly for constant low EV production (loEV, 2.1%). EVs increase the overall virus loss rate (= outflow and EV adsorption), and to compensate, the virus gain rate (= host infection) increases via an increased host concentration. The decrease in host growth rate due to EV production does not affect its concentration because the host concentration is controlled in a top-down manner (rather, it further reduces the virus concentration).

The effect is much more pronounced for oscillating conditions (Fig. 1B). Although the average host concentration is only slightly lower for constant low EV production (−2.6%), the oscillation amplitude is substantially reduced. Simulations with constant high EV (hiEV) and regulated EV (regEV) production generally eliminate oscillations throughout much of the parameter space (range in inflow concentrations and dilution rates).

Note that these are true predictions or emergent output from the models. In other words, no parameters were adjusted/calibrated to make EVs beneficial.

The dampening effect of EV production can be explained in layman’s terms considering a simple predator–prey, i.e. Lotka–Volterra, interaction. Without EV production, starting with relatively high cells and low virus, the virus has plenty of “food” and increases, which results in high cell and high virus. Then the virus starts to affect the cell concentration, which leads to low cell and still high virus. Then the low cell means the virus has a lower growth rate, which leads to low cell and low virus. Then, the lower virus allows the cell to grow, which leads to high cell and low virus, and so on. When EVs are produced, their concentration generally follows the same pattern as cells, so they act to slow down the virus at the time when it otherwise would grow fast, thus dampening the oscillation. For regulated EV production (regEV), this effect is even more pronounced.

*Without EV:* high cells, low virus → high cells, high virus → low cells, high virus → low cells, low virus → high cells, low virus → …

*With EV:* high cells $\color{red}{\& \ \mathrm{EV}}$, low virus → high cells $\color{red}{\& \ \mathrm{EV}}$, somewhat high virus → somewhat low cells $\color{red}{\& \ \mathrm{EV}}$, somewhat high virus → somewhat low cells $\color{red}{\& \ \mathrm{EV}}$, low virus → high cells $\color{red}{\& \ \mathrm{EV}}$, low virus → …

Quantifying the fitness advantage of one strain over another or the benefit of a specific trait is a common task but not entirely straightforward in this case, and some discussion is appropriate here. Common methods include comparing growth rates in batch culture, minimum resource requirements (i.e. R*; Tilman 1982), or competition/invasion experiments. However, none of these are suitable here for the following reasons. Growth rates do not quantify the effect of viral grazing, i.e. reducing virus concentrations with EVs may affect fitness even for the same growth rate. R* is for resource-limited growth, but viruses introduce a top-down component (already mentioned above). Competition experiments would either result in coexistence when viruses are strain-specific (i.e. Kill-the-Winner mechanism; Thingstad 2000) or cheating when they are general. In these simulations, the most striking effect of EV production is to reduce oscillations, and this can be considered to be the main benefit. From an evolutionary perspective, strong oscillations mean lower minimum concentrations and higher extinction risk.

### EVs expand the existence space in L77 model

**Figure 2.**
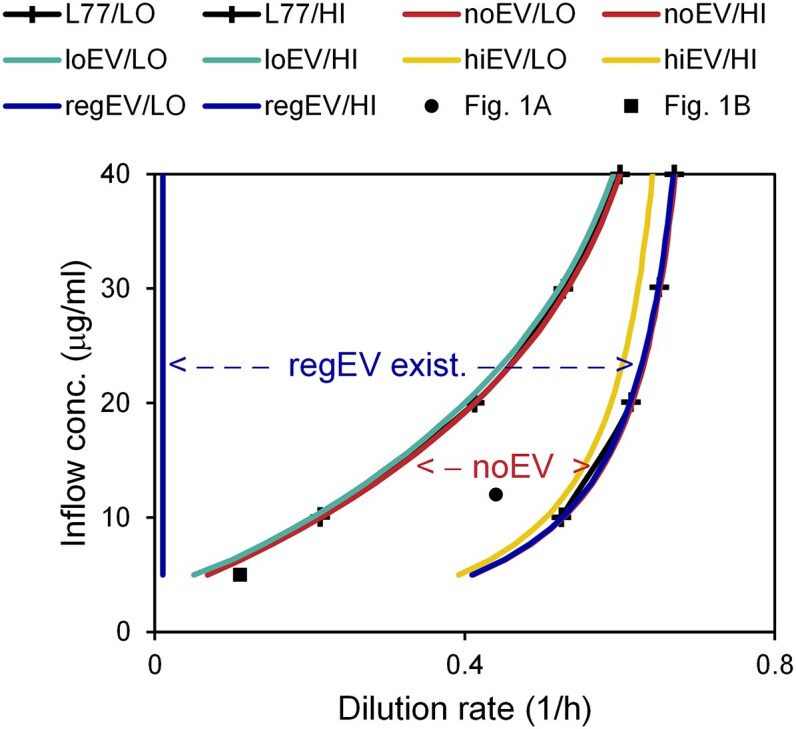
Existence space of host for L77 *E. coli* model. Various dilution rates (*ρ*) and inflow substrate concentrations (*C*). Compare to Fig. 4 in L77. For L77/*Pro*, see Supplementary Fig. S4.

The host existence space, defined as the minimum concentration above a threshold (*n* > 10 no./ml, as L77), is bounded at low dilution rates by strong virus loss and oscillations to extinction and at high dilution rates by negligible virus and washout (Fig. 2). Simulations without virus result in an identical upper bound (not shown).

Constant low EV production shifts the existence space slightly toward lower dilution rates (loEV; the higher bound is covered by the green regEV line). The higher bound is defined by washout with a negligible effect of the virus, which occurs at a slightly lower dilution rate for the strain with EV production due to the additional resources spent (see Equation 1). The lower bound is controlled by virus predation, and the strain with constant low EV production can extend that slightly by dampening oscillations (see Fig. 1B). The simulation with constant high EV production (hiEV) has a stronger shift, and it even extends the existence space down to zero dilution rate. This high EV production completely eliminates oscillations, and the host can survive, despite substantial virus loss. The simulation with regulated EV production shows low cost at high dilution rates (EV production is down-regulated) and elimination of oscillations at low dilution rates (EV production is upregulated), thus it exhibits “the best of both worlds”. Similar results are obtained for the L77 model with *Pro* (see Supplementary Fig. S4).

### EVs dampen or eliminate oscillations in all models

**Figure 3.**
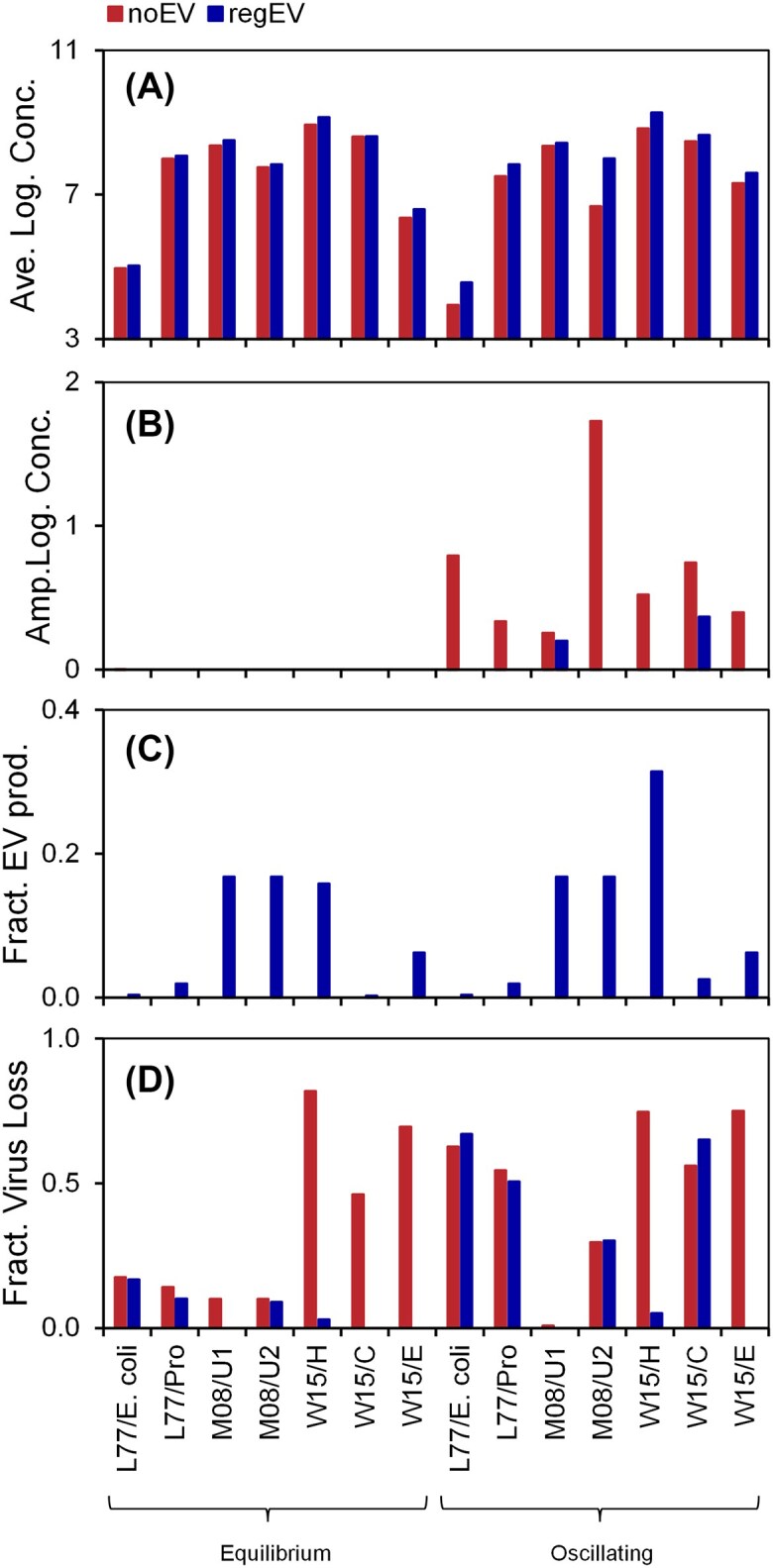
Effect of EV production on (A) average host concentration, (B) amplitude of host concentrations, (C) fraction of resources spent on EV production, and (D) fraction of lost to viral predation. Results from models without EV production (noEV) and regulated EV production (regEV). All values for uninfected cells.

A comparison of all models for simulations without EV production (noEV) and regulated EV production (regEV) for two sample equilibrium and oscillating conditions shows a consistent picture (Fig. 3). The average host concentration is relatively unaffected by EV production (average increase in log concentration = +4.3%), but oscillations are dampened or completely eliminated.

Here, models without and with EV production are compared using the original parameterization. An alternative approach is to adjust the parameters to account for the EV production. For example, the maximum host growth rate in the L77 model is based on experiments, but those presumably included EV production. Therefore, EV production should not lower the rate as shown in Equation (1). A more appropriate analysis may be to compare simulations with a higher maximum growth rate (i.e. *μ* times [1 + *f_R_ n*_EV_] in Equation 1), so that the strain with EV production has a growth rate corresponding to the experimental values. The same issue applies to the viruses, where the loss process presumably always included adsorption to EVs. To explore this, all simulations were repeated with an “alternate parametrization”, including increased maximum growth rate and decreased virus loss rate (details in supplementary data). Some of these simulations are substantially different (e.g. the eukaryotes in the W15 model do not survive without EV production), but they generally produce consistent results, i.e. they do not change the main conclusion (see Supplementary Fig. S7).

### Comparison to field observations

**Figure 4.**
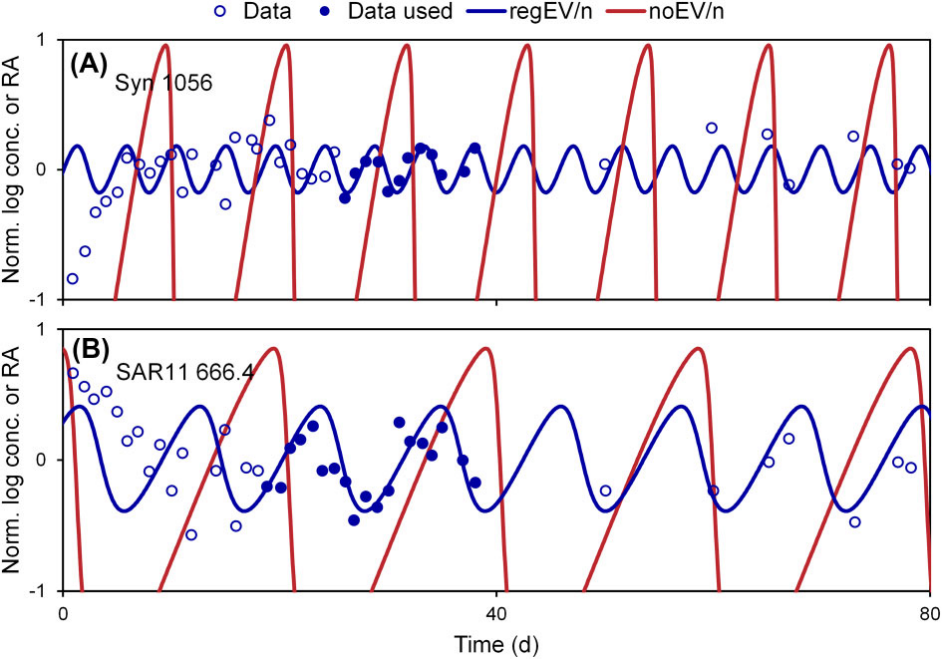
Without EVs, ecosystems would exhibit stronger oscillations. The L77 model with regulated EV production was calibrated to the observations of Needham et al. (2013), and then EV production was turned off. Data normalized to data mean.

The models suggest that EVs dampen oscillations, or that (given EVs are produced) oscillations would be stronger if EVs were not produced. To explore this, the L77 model with regulated EV production (regEV) was calibrated to time series observations of a marine cyanobacteria (*Synechococcus*) and heterotrophic bacteria (SAR11) (Needham et al. 2013), and then the EV production was turned off (Fig. 4). The model predicts that, for these two species, if EVs were not produced, the amplitude of oscillations would be higher by factors of 5100, and 83, respectively.

### Effect of EVs on carbon fluxes

EVs alter the carbon flow in the ecosystem. For the organisms and environmental conditions evaluated here, EV production constitutes 10% of productivity (average across all simulations with regulated EV production) (Fig. 3C). The EVs also reduce the viral lysis fraction (ratio of virus loss to total loss rate), on average from 43% to 18% (Fig. 3D). How do these predictions relate to existing estimates of carbon cycling in the ocean?

Excretion and exudation by phytoplankton (dissolved primary production) is estimated to be around 25% of primary production (Moran et al. 2022). A first thought may be that this number needs to be increased by 10% to account for EV production. However, existing estimates based on separating cells by filtration include EVs as part of the dissolved fraction (i.e. size < 0.2 μm). Therefore, EV production can be considered a component of this flux. In other words, the EV production was already always a part of the exudation and excretion measurements. However, EVs are not what is normally considered as dissolved organic matter (DOM), including various molecules like amino acids or polysaccharides. Therefore, EV production does not alter understanding of *how much* is produced, but *what* is produced.

Estimates for phytoplankton and bacteria viral lysis, aka the “viral shunt”, are around 10%–50% or 6%–26% of primary production (Fuhrman 1999, Wilhelm and Suttle 1999). The model values for with and without EVs fall within this range but suggest a substantial reduction due to EVs. Again, an initial thought may be that this estimate would need to be adjusted down based on the findings here. However, those calculations are typically done by piecing together several observations and calculations, which again already include the effect of vesicles. First, viral loss rate estimates from laboratory data already include adsorption to EVs, which would not be removed when preparing a cell-free fraction by filtration. Second, these calculations often use observations of infected cells (versus infection rate), so they are immune (no pun intended) to the explicit consideration of EVs. In other words, estimates of the viral shunt are based on successful infection, which is equal to the adsorption rate multiplied by virus concentration. Here, the former parameter is poorly constrained. If the virus concentration is lower due to EV adsorption, then the adsorption rate just has to be that much higher. Therefore, EV production does not alter estimates of the viral shunt, but it does add another viral loss mechanism.

## Summary and outlook

Here, the effectiveness of EVs as viral countermeasures was explored. Extending a number of existing models showed that the fitness effect of EV production depends on the environmental conditions and production rate. Constant low production (loEV) has little effect. Constant high production (hiEV) dampens predator–prey oscillations but reduces cell concentration when virus loss is low. Regulated production (regEV), the most realistic scenario, also dampens oscillations and does not reduce cell concentration substantially. In the context of evolution, dampening oscillations increases the minimum cell concentration and reduces the risk of extinction, and thus provides a fitness benefit.

In the natural/ambient environment, oscillations are likely to be modified and dampened by other factors, and less pronounced than in the idealized model environments simulated here. However, environmental observations do show substantial oscillations (Needham et al. 2013) and EV production (Biller et al. 2014, Silva et al. 2022), so damping by EVs is likely to be important in the microbial ecology of natural systems.

It would be useful to confirm these model predictions experimentally. This could be done, e.g. using laboratory cultures with bacteria and viruses (Levin et al. 1977), including wild-type and vesicle production mutants (Manning and Kuehn 2011).

This work serves as a first attempt/step to understand the role of EVs as viral countermeasures and integrate them into ecological models. Future models may (a) extend the EV production sub-model to include various environmental factors (Biller et al. 2023), (b) consider additional ecological functions, like transport of hydrolytic enzymes (Biller et al. 2022, Fadeev et al. 2022, Li et al. 2024), which can build on other existing models, incl. those that consider cheating (Allison 2005), and (c) up-scale by incorporating into ecosystem-scale models (Butenschön et al. 2016).

## Supplementary Material

fiaf030_Supplemental_File
