# Supplementary material for: Extracellular vesicles as viral countermeasures: dampening of oscillations and reduction of extinction risk
Source: FEMS Microbiol Ecol. 2025 Apr 12;101(5):fiaf030. doi: 10.1093/femsec/fiaf030 (PMC11995696; doi:10.1093/femsec/fiaf030)
Supplement: fiaf030_Supplemental_File [file fiaf030_supplemental_file.docx]

**Extracellular vesicles as viral countermeasures: Dampening of oscillations and reduction of extinction risk**

Ferdi L. Hellweger (1)*

(1) Water Quality Engineering, Technical University of Berlin, Berlin, Germany

*Correspondence: ferdi.hellweger@tu-berlin.de

**Supplementary Information**

**Contents**

S1. Model details

S2. Additional results

S3. References

**S1. Model details**

The model codes are available from the author’s GitHub page: <https://github.com/fhellweger/Vesicles>. The distribution includes a Readme.docx file with instructions.

S1a. L77 model

- State variables, equations and parameters are listed in Tables S1 – S3. Parts added for the simulation of EVs are shown in red text.
- The model (without EVs, noEV) was benchmarked against that of (Levin et al 1977) by comparing to Fig. 4 of the reference, as shown in Fig. 2 here.
- The EV production rate for uninfected cells (*n_EV,n_*) was set to the minimum population-level rate (*n_EV,min_*). The EV production rate for infected cells (*n_EV,m_*) was set to the maximum population-level rate (*n_EV,reg_*), increased by a factor to account for the fraction of infected cells.
- The original model was set up for *E. coli* and an additional parameterization for *Pro* was developed here.
- For the “alternate parameterization” (see main text), *φ* is multiplied by [1 + *f_R_* *n_EV,n_*] and *Q*/*V* in the virus mass balance is multiplied by [1 – *f_A_* *γ* ave(*n*) *n_EV,n_*].
- For the application to the (Needham et al 2013) time series observations (Fig. 4):
  - 2-3 cycles that exhibited oscillations were identified visually.
  - A sine curve was fitted manually to those data.
  - The regEV model was calibrated to the resulting period and amplitude.

**Table S1. L77 model state variables (a)**

|  | ***E. coli*** | ***Pro*** |  |
| --- | --- | --- | --- |
| **Symbol** | **Units** | **Units** | **Description** |
| *r* | μg/ml | μmolN/L | Resource |
| *n* | no./ml | no./L | Clean microbe |
| *m* | no./ml | no./L | Infected microbe |
| *v* | no./ml | no./L | EV |
| *w* | no./ml | no./L | “Infected” EV |
| *p* | no./ml | no./L | Virus |

(a) Red text: modifications introduced here. Blue text: Calculated parameters.

**Table S2. L77 model equations (a)**

| **Equation** | **No.** |
| --- | --- |
| $\frac{dr}{dt}=\rho\left( C-r \right)-\phi\left( n+m \right)$ | 1.1 |
| $\frac{dn}{dt}=n \frac{\phi}{e \left( 1+f_{R} n_{EV,n} \right)}-\rho n-\gamma n p$ | 1.2 |
| $\frac{dm}{dt}=\gamma n p-\rho m-e^{-\rho l} \gamma n_{t-l} p_{t-l}$ | 1.3 |
| $\frac{dv}{dt}=n \frac{\phi}{e \left( 1+f_{R} n_{EV,n} \right)} n_{EV,n}+m \frac{\phi}{e \left( 1+f_{R} n_{EV,m} \right)} n_{EV,m}-\rho v-f_{A} \gamma v p$ | 1.4 |
| $\frac{dw}{dt}=f_{A} \gamma v p-\rho w$ | 1.5 |
| $\frac{dp}{dt}=b e^{-\rho l} \gamma n_{t-l} p_{t-l}-\rho p-\gamma n p-f_{A} \gamma v p$ | 1.6 |
| $\phi=P \frac{r}{Q+r}$ | 1.7 |
| $f_{VL}=\frac{\gamma p}{\rho+\gamma p}$ | 1.8 |

(a) See Table S1, footnote (a).

**Table S3. L77 model input parameters (a)**

|  | ***E. coli*** |  | ***Pro*** |  |  |  |
| --- | --- | --- | --- | --- | --- | --- |
| **Symbol** | **Units** | **Value (b)** | **Units** | **Value *Pro*** | ***Syn* (q)** | **SAR11 (q)** |
| *Model parameters* |  |  |  |  |  |  |
| *ρ* | 1/h | 0.45 (0.20-0.70) | 1/d | 0.50 (f) | 2.0 | 1.0 |
| *C* | μg/ml | 25 (10-40) | μmolN/L | 0.80 (g) | 0.80 | 0.80 |
| *e* | μg/no. | 1.35e-5 | μmolN/no. | 1.1e-9 (h) | 1.2e-9 | 1.3e-9 |
| *f_R_* | - | 9.0e-4 | - | 5.4e-3 (i) | 5.4e-3 | 5.4e-3 |
| *n_EV,n_* | - | = *n_EV,min_* | - | = *n_EV,min_* | = *n_EV,min_* | = *n_EV,min_* |
| *n_EV,m_* | - | = *n_EV,reg_* × 16 (d) | - | = *n_EV,reg_* × 14 (d) | = *n_EV,reg_* × 2.2 | = *n_EV,reg_* × 1.2 |
| *γ* | ml/no./h | 6.24e-8 | L/no./d | 1.5e-10 (j) | 9.4e-10 | 4.7e-10 |
| *f_A_* | - | 0.33 | - | 0.66 (k) | 0.66 | 0.66 |
| *b* | - | 98 | - | 45 (l) | 73 | 73 |
| *l* | h | 0.5 | d | 0.71 (l) | 0.080 | 0.16 |
| *P* | μg/no./h | 9.96e-6 | μmolN/no./d | 1.1e-9 (m) | 5.9e-9 | 3.0e-9 |
| *Q* | μg/ml | 4.0 | μmolN/L | 0.33 (n) | 0.45 | 0.45 |
| *Other parameters* |  |  |  |  |  |  |
| *d_cell_* | μm | 1.5 (e) | μm | 0.65 (e) | - | - |
| *d_EV_* | nm | 75 (o) | nm | 75 (o) | - | - |
| *d_virus_* | nm | 49 (p) | nm | 50 (p) | - | - |
| *n_EV,min_* | - | 4.0 (c) | - | 3.5 (c) | 1.7 | 0.88 |
| *n_EV,reg_* | - | 91 (c) | - | 44 (c) | 44 | 44 |

(a) See Table S1, footnote (a).

(b) Basecase (range). Parameters for original model based on Figs. 1 and 4 in (Levin et al 1977).

(c) See main text. For *E. coli*, an up-regulated release rate is taken as 23, based on species-specific observations.

(d) Increased to account for the ratio of total to infected cells, average of noEV simulations.

(e) *E. coli*: Based on cell volume of 1.8 μm^3^, based on 0.1 – 3.5 μm^3^ (Loferer-Krößbacher et al 1998), assuming spherical geometry. *Pro*: Based on 0.5-0.8 μm (Bertilsson et al 2003).

(f) = *μ* = 0.5 1/d (Kirchman 2016).

(g) Set to get *n* = ~3.0e4 cells/ml = ~3.0e7 cells/L, upper 50m at BATS (Lomas et al 2022).

(h) Corresponds to 7.0 fmolC/cell, based on 4.1 – 10 fmolC/cell, (Partensky et al 1999), converted using 16 molN / 106 molC (Redfield).

(i) Calculation:

Fraction of resources required for membrane (*f_MEM_*) = 0.33, based Ortega-Arzola et al (2024) (Table 1, lipid bilayer + 30% proteome, average of mass (g/100 g Cells) and energy (J/cell)).

*f_R_* = (1-*f_MEM_*) × *V_EV_* / *V_cell_* + *f_MEM_* × *A_EV_* / *A_cell_* = (1-*f_MEM_*) × (*d_EV_* / *d_cell_*)^3^ + *f_MEM_* × (*d_EV_* / *d_cell_*)^2^ = see table entry.

(j) Set to get *p* = ~10 *n*, based on VBR = 3-20 (Parsons et al 2012).

(k) Factor accounts for diffusive encounters (β) and attachment probability (α).

Calculation:

Diffusive encounters: Based on Słomka et al (2023) (Eq. 3.1 & associated text). *a* equiv. *r*.

*f_β_* = *Γ_diff,p,v_* / *Γ_diff,p,n_* = [(1/*a_p_* + 1/*a_v_*) × (*a_p_* + *a_v_*)] / [(1/*a_p_* + 1/*a_n_* ) × (*a_p_* + *a_n_*)].

Near-field interactions: Based on Elimelech et al (1995) (*V_T_* = *V_R_* + *V_A_* in Tables 3.2 and 3.3 proportional to size) and Stumm and Morgan (1996), (*W* in Table A14.1 proportional to *V_T_* and size), *a* equiv. *a_1_* × *a_2_*/(*a_1_* + *a_2_*).

*f_α_* = (1/*W_p,v_*) / (1/*W_p,n_*) = *W_p,n_* / *W_p,v_* = [*a_p_* × *a_n_* / (*a_p_* + *a_n_*)]^2^ / [*a_p_* × *a_v_* / (*a_p_* + *a_v_*)]^2^ = 2.22.

Overall: *f_A_* = *f_β_* × *f_α_* = see table entry.

(l) From Table 3 in (Laurenceau et al 2021), burst size is upper bound, see reference.

(m) Corresponds to *μ_max_* (= *P* / *e*) = 2 *μ*.

(n) Upper 50 m at Bermuda, BATS core data.

(o) Based on (Biller et al 2014).

(p) *E. coli*: See reference for M08. *Pro*: Lindell et al (2004).

(q) Values used for the applications to observations from (Needham et al 2013) presented in Fig. 4.

S1b. M08 Model

- The benchmark results are presented in Fig. S1.
- Bacteria size, needed for *f_R_* and *f_A_* calculation, is based on M08 (*c_B_*). However, virus size in M08 was not based on published values and is therefore assigned based on other sources.
- Regulation of EV production is implemented as in L77.
- Assumed the decay rate and regeneration ratio for EVs is same as for virus (*r_V_*, *λ*).
- The equilibrium scenario corresponds to the default parameter set of M08. For the oscillating scenario, several parameters were changed (see Table S6). This parameter set was determined by a parameter perturbation analysis, where parameters were increased/decreased by a factor of 2 to obtain oscillating results. For those cases that result in oscillation, the model with EV production consistently exhibited a lower amplitude in host concentration. Only one representative case is adopted here.
- For the “alternate parameterization” (see main text), *α_U_* is multiplied by [1 + *f_R_* *n_EV,U_*] (also for *Y* and *R*) and *λ* is multiplied by [1 – *f_A_* *β* ave(*U*) *n_EV,U_*].

Fig. S1. Benchmark of M08 model. Corresponds to M08 Table 3. (A) BC1, (B) BC2, (C) Carbon flow to protozoans.

**Table S4. M08 model state variables (a)**

| **Symbol** | **Units** | **Description** |
| --- | --- | --- |
| *U* | no./L | Uninfected bacteria |
| *I* | no./L | Infected bacteria |
| *Y* | no./L | Lysogenic bacteria |
| *R* | no./L | Resistant bacteria |
| *O* | no./L | EVs |
| *V* | no./L | Viruses |
| *P* | no./L | Protozoans |
| *C* | μgC/L | Organic carbon resource |

(a) See Table S1, footnote (a).

**Table S5. M08 model equations (a)**

| **Equation** | **No.** |
| --- | --- |
| $\frac{dU_{j}}{dt}={c_{B}}^{-1} e_{B} \alpha_{U} C_{j} \frac{1}{1+f_{R} n_{EV,U}}U_{j}-\beta V_{j} U_{j}-\alpha_{PU} P U_{j}-D U_{j}$ | 2.1 |
| $\frac{dI_{j}}{dt}=\beta V_{j} U_{j}-\alpha_{PI} P I_{j}-m_{L} I_{j}-a I_{j}+b Y_{j}-D I_{j}$ | 2.2 |
| $\frac{dY_{j}}{dt}=\left( n_{V} c_{V}+c_{B} \right)^{-1} e_{B} \alpha_{Y} C_{j} \frac{1}{1+f_{R} n_{EV,Y}} Y_{j}-\alpha_{PY} P Y_{j}+a I_{j}-b Y_{j}-D Y_{j}$ | 2.3 |
| $\frac{dR_{j}}{dt}={c_{B}}^{-1} e_{B} \alpha_{R} C_{j} \frac{1}{1+f_{R} n_{EV,R}}R_{j}-\alpha_{PR} P R_{j}-D R_{j}$ | 2.4 |
| $\frac{dO_{j}}{dt}={c_{B}}^{-1} e_{B} \alpha_{U} C_{j} \frac{1}{1+f_{R} n_{EV,U}} n_{EV,U} U_{j}+{c_{B}}^{-1} e_{B} \alpha_{U} C_{j} \frac{1}{1+f_{R} n_{EV,I}} n_{EV,I} I_{j}+\left( n_{V} c_{V}+c_{B} \right)^{-1} e_{B} \alpha_{Y} C_{j} \frac{1}{1+f_{R} n_{EV,Y}} n_{EV,Y} Y_{j}+{c_{B}}^{-1} e_{B} \alpha_{R} C_{j} \frac{1}{1+f_{R} n_{EV,R}} n_{EV,R} R_{j}-f_{A} \beta V_{j} O_{j}-\lambda O_{j}-D O_{j}$ | 2.5 |
| $\frac{dV_{j}}{dt}=e_{V} \left( {c_{B}}/{c_{V}}+n_{V} \right) m_{L} I_{j}-n_{V} \beta V_{j} U_{j}-f_{A} \beta V_{j} O_{j}-\lambda V_{j}-D V_{j}$ | 2.6 |
| $\frac{dP}{dt}={c_{P}}^{-1} e_{P} \left[ c_{B} \left( \alpha_{PU} U_{1}+\alpha_{PU} U_{2}+\alpha_{PR} R_{1}+\alpha_{PR} R_{2} \right)+\left( n_{V} c_{V}+c_{B} \right)\left( \alpha_{PI} I_{1}+\alpha_{PI} I_{2}+\alpha_{PY} Y_{1}+\alpha_{PY} Y_{2} \right) \right] P-m_{P} P-D P$ | 2.7 |
| $\frac{dC_{1}}{dt}=s_{1}-\left( \alpha_{U} U_{1}+\alpha_{Y} Y_{1}+\alpha_{R} R_{1} \right) C_{1}-D C_{1}+r_{V} \left[ \left( 1-e_{V} \right) \left( n_{V} c_{V}+c_{B} \right) m_{L} \left( I_{1}+I_{2} \right)+c_{V} \lambda\left( V_{1}+V_{2} \right)+c_{O} \lambda\left( O_{1}+O_{2} \right) \right]+r_{P} \left[ \left( 1-e_{P} \right) \left\{ c_{B} \left( \alpha_{PU} U_{1}+\alpha_{PU} U_{2}+\alpha_{PR} R_{1}+\alpha_{PR} R_{2} \right)+\left( n_{V} c_{V}+c_{B} \right)\left( \alpha_{PI} I_{1}+\alpha_{PI} I_{2}+\alpha_{PY} Y_{1}+\alpha_{PY} Y_{2} \right) \right\} P+m_{P} c_{P} P \right]$(b) | 2.8 |
| $\frac{dC_{2}}{dt}=s_{2}-\left( \alpha_{U} U_{2}+\alpha_{Y} Y_{2}+\alpha_{R} R_{2} \right) C_{2}-D C_{2}$ | 2.9 |
| $f_{VL,j}={\beta V_{j} U_{j}}/\left( \beta V_{j} U_{j}+\alpha_{PU} P U_{j}+D U_{j} \right)$ | 2.10 |

(a) See Table S1, footnote (a).

(b) Green term is given as *c_B_* in original model documentation, assumed typo.

**Table S6. M08 model input parameters (a)**

|  |  | **Value** |  |
| --- | --- | --- | --- |
| **Symbol** | **Units** | **EQU (b)** | **OSC** |
| *Model parameters* |  |  |  |
| *α_U_* | L/day/no. | 2e-10 | ×2 |
| *α_Y_* | L/day/no. | 0.9×*α_U_* | - |
| *α_R_* | L/day/no. | 0.9×*α_U_* | - |
| *f_R_* | - | 6.6e-3 (c) | - |
| *n_EV,U,Y,R_* | - | *= n_EV,min_* | - |
| *n_EV,I_* | - | *= n_EV,reg_* × 100 (f) | - |
| *α_PU_* | L/day/no. | 1e-7 | - |
| *α_PI_* | L/day/no. | *α_PU_* | - |
| *α_PY_* | L/day/no. | *α_PU_* | - |
| *α_PR_* | L/day/no. | *α_PU_* | - |
| *β_1_* | L/day/no. | 5e-11 | - |
| *β_2_* | L/day/no. | 2e-10 | - |
| *f_A_* | - | 0.40 (c) | - |
| *a* | 1/day | 0.1 | - |
| *b* | 1/day | 0.1 | - |
| *m_L_* | 1/day | 3.0 | - |
| *m_P_* | 1/day | 0.1 | - |
| *λ* | 1/day | 0.5 | - |
| *D* | 1/day | 0.01 | - |
| *e_B_* | - | 0.2 | - |
| *e_V_* | - | 0.5 | - |
| *e_P_* | - | 0.3 | - |
| *c_B_* | μgC/no. | 2e-8 | - |
| *c_V_* | μgC/no. | 0.01×*c_B_* | - |
| *c_O_* | μgC/no. | 16×*c_V_* (g) | - |
| *c_P_* | μgC/no. | 100×*c_B_* | - |
| *n_V_* | - | 1.0 | - |
| *r_V_* | - | 0.9 | - |
| *r_P_* | - | 0.1 | - |
| *s_1_* | μgC/L/day | 10.0 | - |
| *s_2_* | μgC/L/day | 5.0 | ×2 |
| *Other parameters* |  |  |  |
| *d_cell_* | μm | 0.98 (h) | - |
| *d_EV_* | nm | 120 (d) | - |
| *d_virus_* | nm | 49 (i) | - |
| *n_EV,min_* | - | 25 (d) | - |
| *n_EV,reg_* | - | 320 (e) | - |

(a) See Table S1, footnote (a).

(b) Table 2 in M08.

(c) Calculation as *Pro*, see Table S3.

(d) Average of heterotrophic bacteria, Biller et al (2023), Table 1.

(e) *n_EV,min_* × 13, see main text.

(f) Increased to account for the ratio of total to infected cells, average of noEV simulations.

(g) Volume ratio for EV and virus.

(h) Based on M08 carbon content of bacteria (*c_B_*), converted assuming 10% DW and 40%C.

(i) See main text.

S1c. W15 Model

- The model (without EVs) was benchmarked against that of (Weitz et al 2015) by comparing to values from Fig. S1 of W15, including attracting (ATT) and limit cycle (LC) conditions (Fig. S2). Overall there is good agreement between the two models. However, there are some differences, e.g. E for LC/W15 vs. LC/noEV. These discrepancies may be due to uncertainties in the initial and final values since they were digitized manually from the figure in W15, and differences in the numerical integration methods used.
- Modifications:
  - Regulation is based on the fraction of virus infection. To account for the regulation response time and dampen out oscillations, a running average is used. Specifically, a regulatory history memory variable (*f_in,a_*) is added, which tracks the infection fraction history using an exponential decaying average. *α_fin_* (d^-1^) is a weighting parameter that characterizes the length of memory. Larger values will give more weight to the current value (less memory) and smaller values will give more weight to the historical value (more memory). It is set proportional to the growth rate.
  - EVs degrade (Eq. 3.5+) to Organic N (Eq. 3.11)
  - Viruses “infect” EVs, which removes viruses and vesicles (Eq. 3.8) and adds the mass to Organic N (Eq. 3.5).
- The equilibrium scenario corresponds to the ATT parameter set of W15. The LC parameter set of W15 did not result in long-term oscillations in our implementation of the model. Therefore, for the oscillating scenario, a number of parameters were changed (see Table S9). This parameter set was determined by a perturbation analysis, where parameters were increased/decreased by a factor of 2 to obtain oscillating results. For those cases that result in oscillation, the model with EV production consistently exhibited a lower amplitude in host concentration. Only one representative case is adopted here.
- For the “alternate parameterization” (see main text), *μ_H_* is multiplied by [1 + *f_R,H_* *n_EV,H_*] and *m_VH_* is multiplied by [1 – *f_A,H_* *φ_VH_* ave(*H*) *n_EV,H_*] (also for *C* and *E*).

Fig. S2. Benchmark of W15 model. Concentrations at t = 300d.

**Table S7. W15 model state variables (a)**

| **Symbol** | **Units** | **Description** |
| --- | --- | --- |
| *H* | particles/L | Heterotrophs concentration |
| *C* | particles/L | Cyanobacteria concentration |
| *E* | particles/L | Euk. autos concentration |
| *Z* | particles/L | Zooplankton |
| *O_H_* | particles/L | EVs of H concentration |
| *O_C_* | particles/L | EVs of C concentration |
| *O_E_* | particles/L | EVs of E concentration |
| *V_H_* | particles/L | Viruses of H concentration |
| *V_C_* | particles/L | Viruses of C concentration |
| *V_E_* | particles/L | Viruses of E concentration |
| *x_on_* | μmol/L | Organic N concentration |
| *x_in_* | μmol/L | Inorganic N concentration |

(a) See Table S1, footnote (a).

**Table S8. W15 model equations (a)**

| **Equation** | **No.** |
| --- | --- |
| $\frac{dH}{dt}=\mu_{H} \frac{x_{on}}{K_{on}+x_{on}}\frac{1}{1+f_{R,H} n_{EV,H}} H-\phi_{VH} H V_{H}-\psi_{ZH} H Z-m_{on,H} H-m_{in,H} H$ | 3.1 |
| $\frac{dC}{dt}=\mu_{C} \frac{x_{in}}{K_{in,C}+x_{in}}\frac{1}{1+f_{R,C} n_{EV,C}} C-\phi_{VC} C V_{C}-\psi_{ZC} C Z-m_{on,C} C-m_{in,C} C$ | 3.2 |
| $\frac{dE}{dt}=\mu_{E} \frac{x_{in}}{K_{in,E}+x_{in}}\frac{1}{1+f_{R,E} n_{EV,E}} E-\phi_{VE} E V_{E}-\psi_{ZE} E Z-m_{on,E} E-m_{in,E} E$ | 3.3 |
| $\frac{dZ}{dt}=p_{g}\left( \frac{q_{H}}{q_{Z}} \psi_{ZH} H+\frac{q_{C}}{q_{Z}} \psi_{ZC} C+\frac{q_{E}}{q_{Z}} \psi_{ZE} E \right) Z-m_{Z} Z-m_{ZP} Z^{2}$ | 3.4 |
| $\frac{dO_{H}}{dt}=\mu_{H} \frac{x_{on}}{K_{on}+x_{on}}\frac{1}{1+f_{R,H} n_{EV,H}} n_{EV,H} H-f_{A,H} \phi_{VH} O_{H} V_{H}-m_{O,H} O_{H}$ | 3.5 |
| $\frac{dO_{C}}{dt}=\mu_{C} \frac{x_{in}}{K_{in,C}+x_{in}}\frac{1}{1+f_{R,C} n_{EV,C}} n_{EV,C} C-f_{A,C} \phi_{VC} O_{C} V_{C}-m_{O,C} O_{C}$ | 3.6 |
| $\frac{dO_{E}}{dt}=\mu_{E} \frac{x_{in}}{K_{in,E}+x_{in}}\frac{1}{1+f_{R,E} n_{EV,E}} n_{EV,E} E-f_{A,E} \phi_{VE} O_{E} V_{E}-m_{O,E} O_{E}$ | 3.7 |
| $\frac{dV_{H}}{dt}=\beta_{H} \phi_{VH} H V_{H}-f_{A,H} \phi_{VH} O_{H} V_{H}-m_{VH} V_{H}$ | 3.8 |
| $\frac{dV_{C}}{dt}=\beta_{C} \phi_{VC} C V_{C}-f_{A,C} \phi_{VC} O_{C} V_{C}-m_{VC} V_{C}$ | 3.9 |
| $\frac{dV_{E}}{dt}=\beta_{E} \phi_{VE} E V_{E}-f_{A,E} \phi_{VE} O_{E} V_{E}-m_{VE} V_{E}$ | 3.10 |
| $\frac{dx_{on}}{dt}=-\frac{q_{H}}{{}_{H}} \mu_{H} \frac{x_{on}}{K_{on}+x_{on}} H+q_{V} m_{VH} V_{H}+q_{V} m_{VC} V_{C}+q_{V} m_{VE} V_{E}$  $+\left( q_{H}-q_{V} \beta_{H} \right) \phi_{VH} H V_{H}+\left( q_{H} f_{R,H}+q_{V} \right) f_{A,H} \phi_{VH} O_{H} V_{H}$  $+\left( q_{C}-q_{V} \beta_{C} \right) \phi_{VC} C V_{C}+\left( q_{C} f_{R,C}+q_{V} \right) f_{A,C} \phi_{VC} O_{C} V_{C}$  $+\left( q_{E}-q_{V} \beta_{E} \right) \phi_{VE} E V_{E}+\left( q_{E} f_{R,E}+q_{V} \right) f_{A,E} \phi_{VE} O_{E} V_{E}$  $+q_{H} m_{on,H} H+q_{H} f_{R,H} m_{O,H} O_{H}$  $+q_{C} m_{on,C} C+q_{C} f_{R,C} m_{O,C} O_{C}$  $+q_{E} m_{on,E} E+q_{E} f_{R,E} m_{O,E} O_{E}$  $+p_{on} q_{H} \psi_{ZH} H Z+p_{on} q_{C} \psi_{ZC} C Z+p_{on} q_{E} \psi_{ZE} E Z$ | 3.11 |
| $\frac{dx_{in}}{dt}=-\omega\left( x_{in}-x_{sub} \right)+\frac{q_{H} \left( 1-{}_{H} \right)}{{}_{H}} \mu_{H} \frac{x_{on}}{K_{on}+x_{on}} H-q_{C} \mu_{C} \frac{x_{in}}{K_{in,C}+x_{in}} C-q_{E} \mu_{E} \frac{x_{in}}{K_{in,E}+x_{in}} E$  $+q_{Z} m_{Z} Z+p_{in} q_{H} \psi_{ZH} H Z+p_{in} q_{C} \psi_{ZC} C Z+p_{in} q_{E} \psi_{ZE} E Z$  $+q_{H} m_{in,H} H+q_{C} m_{in,C} C+q_{E} m_{in,E} E$ | 3.12 |
| $f_{in,C}=\frac{\phi_{VC} V_{C}}{\mu_{C} \frac{x_{in}}{K_{in,C}+x_{in}}}$ ; equiv. for *H* and *E* | 3.13 |
| $\alpha_{fin,C}=\alpha_{fin,X} \mu_{C} \frac{x_{in}}{K_{in,C}+x_{in}}$ ; equiv. for *H* and *E* | 3.14 |
| $f_{in,a,C}^{t}=\left( 1-\alpha_{fin,C} \Delta t \right)f_{in,a,C}^{t-\Delta t}+\alpha_{fin,C} \Delta t {f_{in,C}}^{t}$; equiv. for *H* and *E* | 3.15 |
| $n_{EV,C}=n_{EV,min,C}+\left( n_{EV,reg,C}-n_{EV,min,C} \right) \frac{{f_{in,a,C}}^{nreg}}{{kreg}^{nreg}+{f_{in,a,C}}^{nreg}}$ ; equiv. for *H* and *E* | 3.16 |
| $f_{VL}=\frac{\phi_{VC} V_{C}}{\phi_{VC} V_{C}+\psi_{ZC} Z+m_{on,C}+m_{in,C}}$ ; equiv. for *H* and *E* | 3.17 |

(a) See Table S1, footnote (a).

**Table S9. W15 model input parameters (a)**

|  |  | **Value (b)** |  |  |
| --- | --- | --- | --- | --- |
| **Symbol** | **Units** | **ATT/W15**  **& EQU** | **LC/W15** | **OSC** |
| *Model parameters* |  |  |  |  |
| *μ_H_* | 1/d | 0.51 | 0.61 | - |
| *K_on_* | μmol/L | 0.94 | 0.68 | - |
| *ε_H_* | - | 0.20 | 0.19 | ×2 |
| *μ_C_* | 1/d | 1.97 | 0.68 | - |
| *K_in,C_* | μmol/L | 0.053 | 0.059 | - |
| *f_R,H_* | - | 2.7e-3 (c) | - | - |
| *f_R,C_* | - | 5.7e-4 (c) | - | - |
| *f_R,E_* | - | 2.5e-5 (c) | - | - |
| *n_EV,min,H_* | - | 25 (d) | - | - |
| *n_EV,min,C_* | - | 3.5 (e) | - | - |
| *n_EV,min,E_* | - | 2,500 (f) | - | - |
| *n_EV,reg,H_* | - | 320 (g) | - | - |
| *n_EV,reg,C_* | - | 44 (g) | - | - |
| *n_EV,reg,E_* | - | 19,000 (f) | - | - |
| *kreg* | - | 0.05 (h) | - | - |
| *nreg* | - | 3 (h) | - | - |
| *μ_E_* | 1/d | 7.5 | 5.4 | - |
| *K_in,E_* | μmol/L | 5.4 | 5.8 | - |
| *φ_VH_* | L/virus/d | 8.0e-12 | 4.3e-11 | - |
| *f_A,H_* | - | 0.28 (c) | - | - |
| *φ_VC_* | L/virus/d | 9.9e-11 | 1.9e-11 | - |
| *f_A,C_* | - | 0.19 (c) | - | - |
| *φ_VE_* | L/virus/d | 5.8e-11 | 6.7e-11 | - |
| *f_A,E_* | - | 0.76 (c) | - | - |
| *β_H_* | - | 25 | 15 | - |
| *β_C_* | - | 16 | 82 | - |
| *β_E_* | - | 440 | 370 | - |
| *m_VH_* | 1/d | 0.17 | 0.59 | - |
| *m_VC_* | 1/d | 0.63 | 0.76 | - |
| *m_VE_* | 1/d | 0.058 | 0.56 | - |
| *ψ_ZH_* | L/zoopl/d | 1.9e-6 | 2.2e-5 | - |
| *ψ_ZC_* | L/zoopl/d | 2.1e-5 | 4.2e-5 | - |
| *ψ_ZE_* | L/zoopl/d | 1.5e-6 | 2.8e-5 | - |
| *p_g_* | - | 0.4 | 0.4 | - |
| *p_on_* | - | 0.3 | 0.3 | - |
| *p_in_* | - | 0.3 | 0.3 | - |
| *m_Z_* | 1/d | 0.048 | 0.051 | - |
| *m_ZP_* | L/cells/d | 3.3e-7 | 2.8e-5 | - |
| *ω* | 1/d | 0.016 | 0.077 | - |
| *x_sub_* | μmolN/L | 7.7 | 2.67 | - |
| *x_on,in_* | μmolN/L | - | - | - |
| *q_H_* | μmolN/cell | 8.8e-10 | 1.4e-9 | - |
| *q_C_* | μmolN/cell | 3.9e-9 | 3.0e-9 | - |
| *q_E_* | μmolN/cell | 2.1e-7 | 6.7e-8 | - |
| *q_Z_* | μmolN/cell | 2.3e-4 | 3.9e-4 | - |
| *q_V_* | μmolN/cell | 2.0e-12 | 2.2e-12 | - |
| *m_in,H_* | 1/d | 0.0069 | 0.0067 | - |
| *m_in,C_* | 1/d | 0.0050 | 0.016 | - |
| *m_in,E_* | 1/d | 0.0043 | 0.0023 | ×2 |
| *m_on,H_* | 1/d | 0.014 | 0.034 | - |
| *m_O,H_* | 1/d | = *m_VH_* | - | - |
| *m_on,C_* | 1/d | 0.022 | 0.0063 | - |
| *m_O,C_* | 1/d | = *m_VC_* | - | - |
| *m_on,E_* | 1/d | 0.011 | 0.022 | - |
| *m_O,E_* | 1/d | = *m_VE_* | - | - |
| *Other parameters* |  |  |  |  |
| *d_cell,H_* | μm | 1.6 (i) | - | - |
| *d_cell,C_* | μm | 2.4 (i) | - | - |
| *d_cell,E_* | μm | 8.1 (i) | - | - |
| *d_EV,H_* | nm | 120 (d) | - | - |
| *d_EV,C_* | nm | 98 (e) | - | - |
| *d_EV,E_* | nm | 80 (f) | - | - |
| *d_virus,H_* | nm | 49 (j) | - | - |
| *d_virus,C_* | nm | 49 (j) | - | - |
| *d_virus,E_* | nm | 170 (k) | - | - |

(a) See Table S1, footnote (a).

(b) Table S1 in Weitz et al (2015).

(c) See main text and L77 model table footnote for calculation details.

(d) Average of heterotrophic bacteria, Biller et al (2023), Table 1.

(e) *n_EV_*: As in L77; *d_EV_*: Average of modes for *Pro* (85 nm) and *Syn* (110 nm), (Biller et al 2014).

(f) For *E. huxleyi* from (Schatz et al 2017);

*n_EV,min,E_* = 2,500, Control at 72h, EV = (6+4)×10^9^ no./ml (Fig. 1e), cells = 4×10^6^ no./ml (Fig. 1c);

*n_EV,reg,E_* = 19,000, EhV201 at 72h, EV = (6+13)×10^9^ no./ml (Fig. 1e), cells = 1×10^6^ no./ml (Fig. 1c).

*d_EV_* = 80 nm, Control at 72h (Fig. 1e).

(g) *n_EV,min_* × 13, see main text.

(h) *nreg*, *kreg* parameters were set to down- and up-regulate EV production at low and high virus infection, respectively. *α_fin,X_* set to dampen out oscillations.

(i) Based on W15 nitrogen content (*q*), converted assuming Redfield, 10% DW and 40%C.

(j) See reference for M08.

(k) EhV, 160-180 nm, (Nissimov et al 2012).

**S2. Additional results**

Fig. S3. Example results from L77 *Pro* model. As Fig. 1. (A) Equilibrium. *ρ* = 0.52 1/d, *C* = 0.5 μmolN/L. (B) Oscillating. *ρ* = 0.47 1/d, *C* = 0.5 μmolN/L.

Fig. S4. Existence space of host for L77 *Pro* model. Various dilution rates (*ρ*) and inflow substrate concentrations (*C*).

Fig. S5.1. Example results from M08 model for bacteria 1. (A) Equilibrium. (B) Oscillating. State variable abbreviations in Table S4. Parameters in Table S6.

Fig. S5.2. Example results from M08 model for bacteria 2. (A) Equilibrium. (B) Oscillating. State variable abbreviations in Table S4. Parameters in Table S6.

Fig. S6.1. Example results from W15 model for heterotrophs (H). (A) Equilibrium. (B) Oscillating. State variable abbreviations in Table S7. Parameters in Table S9.

Fig. S6.2. Example results from W15 model for cyanobacteria (C). (A) Equilibrium. (B) Oscillating. State variable abbreviations in Table S7. Parameters in Table S9.

Fig. S6.3. Example results from W15 model for eukaryotes (E). (A) Equilibrium. (B) Oscillating. State variable abbreviations in Table S7. Parameters in Table S9.

Fig. S7. As Fig. 3, but “alternate parameterization”.

**S3. References**

Bertilsson S, Berglund O, Karl DM, Chisholm SW (2003). Elemental composition of marine Prochlorococcus and Synechococcus: Implications for the ecological stoichiometry of the sea. *Limnology and Oceanography* **48:** 1721-1731.

Biller SJ, Schubotz F, Roggensack SE, Thompson AW, Summons RE, Chisholm SW (2014). Bacterial Vesicles in Marine Ecosystems. *Science* **343:** 183-186.

Biller SJ, Coe A, Arellano AA, Dooley K, Silvestri SM, Gong JS *et al* (2023). Environmental and Taxonomic Drivers of Bacterial Extracellular Vesicle Production in Marine Ecosystems. *Applied and Environmental Microbiology* **89:** e00594-00523.

Elimelech M, Gregory J, Jia X, Williams R (1995). Surface interaction potentials. *Particle Deposition & Aggregation: Measurement, Modelling and Simulation; Butterworth-Heinemann: Jordan Hill, Oxford, UK*.

Kirchman DL (2016). Growth Rates of Microbes in the Oceans. *Annual Review of Marine Science* **8:** 285-309.

Laurenceau R, Raho N, Forget M, Arellano AA, Chisholm SW (2021). Frequency of mispackaging of Prochlorococcus DNA by cyanophage. *The ISME Journal* **15:** 129-140.

Levin BR, Stewart FM, Chao L (1977). Resource-Limited Growth, Competition, and Predation: A Model and Experimental Studies with Bacteria and Bacteriophage. *The American Naturalist* **111:** 3-24.

Lindell D, Sullivan MB, Johnson ZI, Tolonen AC, Rohwer F, Chisholm SW (2004). Transfer of photosynthesis genes to and from <i>Prochlorococcus</i> viruses. *Proceedings of the National Academy of Sciences* **101:** 11013-11018.

Loferer-Krößbacher M, Klima J, Psenner R (1998). Determination of Bacterial Cell Dry Mass by Transmission Electron Microscopy and Densitometric Image Analysis. *Applied and Environmental Microbiology* **64:** 688-694.

Lomas MW, Bates NR, Johnson RJ, Steinberg DK, Tanioka T (2022). Adaptive carbon export response to warming in the Sargasso Sea. *Nature Communications* **13:** 1211.

Needham DM, Chow C-ET, Cram JA, Sachdeva R, Parada A, Fuhrman JA (2013). Short-term observations of marine bacterial and viral communities: patterns, connections and resilience. *The ISME Journal* **7:** 1274-1285.

Nissimov JI, Worthy CA, Rooks P, Napier JA, Kimmance SA, Henn MR *et al* (2012). Draft Genome Sequence of Four Coccolithoviruses: Emiliania huxleyi Virus EhV-88, EhV-201, EhV-207, and EhV-208. *Journal of Virology* **86:** 2896-2897.

Ortega-Arzola E, Higgins PM, Cockell CS (2024). The minimum energy required to build a cell. *Scientific Reports* **14:** 5267.

Parsons RJ, Breitbart M, Lomas MW, Carlson CA (2012). Ocean time-series reveals recurring seasonal patterns of virioplankton dynamics in the northwestern Sargasso Sea. *The ISME Journal* **6:** 273-284.

Partensky F, Hess WR, Vaulot D (1999). Prochlorococcus, a Marine Photosynthetic Prokaryote of Global Significance. *Microbiology and Molecular Biology Reviews* **63:** 106-127.

Schatz D, Rosenwasser S, Malitsky S, Wolf SG, Feldmesser E, Vardi A (2017). Communication via extracellular vesicles enhances viral infection of a cosmopolitan alga. *Nature Microbiology* **2:** 1485-1492.

Słomka J, Alcolombri U, Carrara F, Foffi R, Peaudecerf FJ, Zbinden M *et al* (2023). Encounter rates prime interactions between microorganisms. *Interface Focus* **13:** 20220059.

Stumm W, Morgan J (1996). Aquatic chemistry: Chemical equilibria and rates in natural waters, JohnWiley & Sons. *Inc, New York***:** 1022.

Weitz JS, Stock CA, Wilhelm SW, Bourouiba L, Coleman ML, Buchan A *et al* (2015). A multitrophic model to quantify the effects of marine viruses on microbial food webs and ecosystem processes. *The Isme Journal* **9:** 1352.
